# The impact of the COVID-19 pandemic on non-COVID-associated mortality: A descriptive longitudinal study of UK data

**DOI:** 10.1016/j.puhip.2024.100489

**Published:** 2024-03-11

**Authors:** Samuel Makanjuola, Saran Shantikumar

**Affiliations:** Warwick Medical School, England, UK

**Keywords:** COVID-19, SARS-CoV-2, Pandemic, Mortality-rate

## Abstract

**Background:**

It has been previously reported in the literature that the COVID-19 pandemic resulted in overall excess deaths and an increase in non-COVID deaths during the pandemic period.

Specifically, our research elucidates the impact of the COVID-19 pandemic on non-COVID associated mortality.

**Study aim:**

To compare mortality rates in non-COVID conditions before and after the onset of the COVID-19 pandemic in England and Wales.

**Study design:**

Annual mortality data for the years 2011–2019 (pre-pandemic) and 2020 (pandemic) in England and Wales were retrieved from the Office for National Statistics (ONS). These data were filtered by ICD-10 codes for nine conditions with high associated mortality. We calculated mortality numbers – overall and age stratified (20–64 and 65+ years) and rates per 100 000, using annual mid-year population estimates.

**Methods:**

Interrupted time series analyses were conducted using segmented quasi-Poisson regression to identify whether there was a statistically significant change (p < 0.05) in condition-specific death rates following the pandemic onset.

**Results:**

Eight of the nine conditions investigated in this study had significant changes in mortality rate during the pandemic period (2020). All-age mortality rate was significantly increased in: ‘Symptoms Signs and Ill-defined conditions’, ‘Cirrhosis and Other Diseases of the Liver’, and ‘Malignant Neoplasm of the Breast’, whereas ‘Chronic Lower Respiratory Disorders’ saw a significant decrease. Age-stratified analyses also revealed significant increases in the 20–64 age-group in: ‘Cerebrovascular Disorders’, ‘Dementia and Alzheimer's Disease’, and ‘Ischaemic Heart Diseases’.

**Conclusion:**

Trends in non-COVID condition-specific mortality rates from 2011 to 2020 revealed that some non-COVID conditions were disproportionately affected during the pandemic. This may be due to the direct impact COVID-19 had on these conditions or the effect the public health response had on non-COVID risk factor development and condition-related management. Further work is required to understand the reasons behind these disproportionate changes.

## 1 Introduction

COVID-19 is a disease caused by the SARS-CoV-2 virus that was first documented in Wuhan, China in late December 2019 [1]. COVID-19 was subsequently declared a pandemic in March 2020 with the novel virus going on to have significant impacts on global mortality and morbidity [2]. As of January 26, 2022, COVID-19 has resulted in ∼360 million confirmed cases and ∼5.6 million deaths worldwide [3].

Studies have shown there have been both excess years of life lost [4], and excess deaths attributed to the COVID-19 pandemic [5,6], and among high income countries, England and Wales had some of the largest numbers of absolute excess deaths during the pandemic [6]. These excess deaths may have been a direct consequence of the virus and/or an indirect consequence of deaths caused by non-COVID conditions due to the national responses to COVID-19 and the protocols implemented to control the spread of the disease [7].

To prioritise the public health response to COVID-19 in England and Wales, lockdown measures were implemented, healthcare staff were redeployed, and non-emergency operations and routine consultations were postponed. Along with the reluctance of some members of the general public to seek healthcare, there is a risk that the pandemic period provided an environment which fostered suboptimal healthcare access and management of non-COVID medical conditions, particularly in those treated out of hospital.

Assessment of the full impact of the pandemic should include both the direct and indirect consequences for mortality [4], and therefore, discovering whether non-COVID mortality was affected during the pandemic period is essential. Previous studies have shown that there has been an increase in non-COVID deaths during the pandemic compared to what would have otherwise been expected in England and Wales [6]. However, whether specific non-COVID conditions were more gravely affected than others during the pandemic period have yet to be evaluated.

The Office for National Statistics (ONS) regularly releases data on all-cause deaths, including COVID-19 more recently [8]. The aim of this project was to use these data to examine condition-specific mortality trends and assess whether the recorded mortality rates associated with non-COVID conditions have changed since the start of the pandemic.

## 2 Methods

### 2.1 Data collection

This is a retrospective longitudinal study of publicly available data. Annual mortality data for England and Wales are released by Public Health England (PHE) – ONS [8]. It provides annual data on the number of deaths registered by “*age group, sex, year and underlying cause of death, as defined using the International Classification of Diseases, Tenth Revision*” (ICD-10). Counts are given for each unique ICD-10 code and 5-year age-group bracket, for each given year. We used the database released on July 6, 2021, which includes annual data counts for the years 2001–2020. Data from 2021 were not used as we were interested in the immediate step change in mortality rates due to the impact of COVID-19.

A change in the ICD-10 definitions of certain conditions in 2011, along with a lack of a mapping key from pre-2011 to post-2011 codes, meant that we did not consider any of the data from 2001 to 2010 in our research to allow for an accurate comparison and continuity of data.

Mortality data providing the leading causes of death by age-group and sex, for deaths registered in England and Wales was also released by PHE-ONS [8]. From these data we chose to include the following causes of death (with their ICD-10 codes bracketed), as they were the most common: Cerebrovascular Disorders (I60–I69); Chronic Lower Respiratory Disorders (J40-J47); Cirrhosis and other diseases of the Liver (K70–K76); Dementia and Alzheimer's Disease (F01, F03, G30); Ischaemic Heart Diseases (I20–I25); Malignant Neoplasm of Breast (C50); Malignant Neoplasm of Colon Sigmoid (C18–C21); Malignant Neoplasm of Trachea Bronchi (C33–C34); and Symptoms Signs and Ill-defined Conditions (R00-R99).

For each of the chosen ICD-10 code sets (“conditions”), the deaths dataset was filtered using the relevant ICD-10 codes, to calculate the number of annual deaths for each condition across England and Wales, as well as the number of deaths stratified by the age groups 20–64 years, and 65+ years. These age-stratifications were decided upon as existing literature consistently reported that individuals aged 65 and over experienced more severe COVID-19 outcomes, while younger populations suffered lower mortality and morbidity rates [9,10].

Annual mid-year population estimates for England and Wales for the years 2011–2020, by single year of age, were retrieved from the ONS: (https://www.ons.gov.uk/peoplepopulationandcommunity/populationandmigration/populationestimates). This was used to calculate population denominators for all of England and Wales for each included year, as well as population estimates for both chosen age groups of interest. The death data and population data were combined to give a final dataset for analysis.

### 2.2 Data analysis

Annual death rates for each of the chosen conditions were calculated as the number of deaths divided by the population size, and expressed in terms of conditional-specific deaths per 100 000 population each year.

To explore whether there had been a significant change in the rate of condition-specific deaths secondary to the COVID-19 pandemic, interrupted times series (ITS) analyses were conducted using 2011–2019 as the “pre-intervention” and 2020 as the “post-intervention” time periods respectively, with the number of condition-specific deaths per year as the dependent variable and the total annual population as an offset variable to model rates. A dummy variable was used to distinguish between the pre- and post-intervention periods. It is acknowledged that COVID-19 would only have influenced deaths from March 2020, but the lack of monthly datasets meant we had to analyse the entirety of 2020 as being post-COVID. Initial analyses suggested overdispersion of data, so a quasi-Poisson segmental regression model was used. There were no documented missing data in the ONS datasets, and no statistical adjustments were made for potential confounders other than long-term time trend.

All data processing, analyses and visualisations were conducted using the statistical software R, except for age-stratified line graphs which were made using Microsoft Excel. The code written and used for analysis is available from the author upon request.

## 3 Results

The percentage changes in mortality rates between 2019 and 2020 in England and Wales for each of the included conditions, by each of the included age groups, is summarised in Table 1, below.

**Table 1:** **Summary of the percentage change in mortality rates for 2020 versus 2011**–**2019 for included conditions in England and Wales, in total and stratified by age-group, from interrupted time series analyses**. ‘—’ indicates no statistically significant change, 95% confidence intervals are within brackets (.), and p values are in squared brackets [] where p < 0.05 = statistically significant.

	20–64 years	65+ years	All Ages
**Cerebrovascular Disorders**	+9.8% (6.6–14) [0.000751]	__	__
**Chronic Lower Respiratory Disorders**	__	−17% (−23 to −7.4) [0.0092]	−16% (6.8–22) [0.010]
**Cirrhosis and Other Diseases of the Liver**	+15% (8.4–25) [0.00438]	__	+9.4% (3.8–16) [0.01376]
**Dementia and Alzheimer's Disease**	+36% (6.8–94) [0.0483]	__	__
**Ischaemic Heart Diseases**	+11% (6.1–17) [0.00299]	__	__
**Malignant Neoplasm of Breast**	__	+3.8% (1.1–6.7) [0.0273]	+2.5% (0.8–4.4) [0.0237]
**Malignant Neoplasm of Colon Sigmoid**	__	__	__
**Malignant Neoplasm of Trachea Bronchi**	__	−4.1% (−6.5 to −1.5) [0.0172]	__
**Symptoms Signs and Ill-defined Conditions**	__	+18% (11–29) [0.00197]	+17% (11–27) [0.00142]

### 3.1 The effect of the pandemic period on ‘Symptoms Signs and Ill-defined conditions’ (SSIDC)

From the period spanning 2011–2020 there was an increase in the all-age mortality rate associated with SSIDC (Fig. 1a). However, despite the increasing trend there was a further significant step increase in the overall mortality rate from 2019 (pre-pandemic era) to 2020 (pandemic era) coded as SSIDC in all ages than was to be expected using pre-pandemic mortality estimates (Fig. 1b). Age-stratified analyses revealed that in 2020 all-age mortality significantly increased by 17% (95% CI = 11–27, p = 0.001) and 65+ mortality increased by 18% (95% CI = 11–29, p = 0.002), but there was no significant change in mortality rate in the age group 20–64 (Table 1).

**Fig. 1a:**
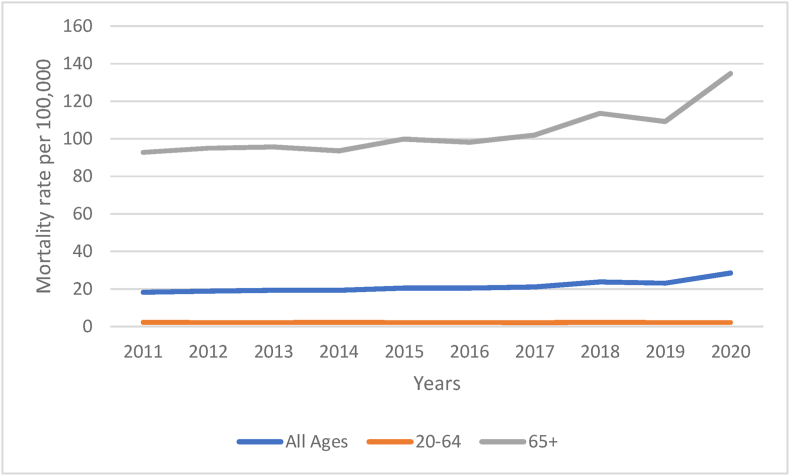
The trend in mortality rates of those that died of ‘Symptoms Signs and Ill-defined Conditions’ in England and Wales stratified by age-group.

**Fig. 1b:**
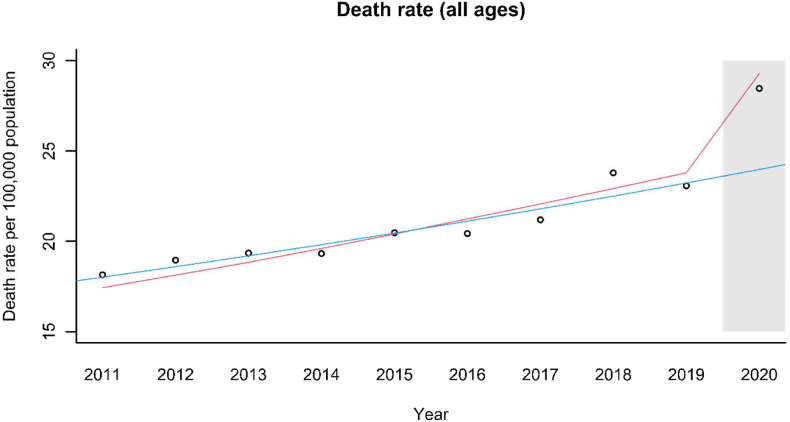
An Interrupted Time Series Analysis (ITSA) of all-age mortality associated with ‘Symptoms Signs and Ill-defined Conditions’ in England and Wales. The grey box indicates the post-COVID period, the blue line is the expected trend without COVID (the counterfactual), and the red line is the true underlying trend.

### 3.2 The effect of the pandemic period on other conditions

There was a significant step increase in the all-age mortality rate from Cirrhosis and Other Diseases of the Liver following COVID-19. This appeared to be driven solely by the 20–64 age-group, which had a mortality increase of 15% (95% CI = 84-25, p = 0.004).

There was an increase in mortality rate from cerebrovascular disorders between 2019 and 2020, which was only statistically significant in the 20–64 year age group (increase of 9.8%, 95% CI = 6.6–14, p < 0.001).

There was a statistically significant decrease in mortality rates from Chronic Lower Respiratory Disorders of 16% in all ages (95% CI = 6.8–22, p = 0.010) after considering the pre-pandemic time trend. Age-stratified analyses found a significant decrease of 17% (95% CI = 7.4–23, p = 0.009) in the 65+ age-group, whereas the 20–64 age-group showed statistically non-significant changes (Table 1).

Malignant Neoplasms of the Breast showed a significant increase in mortality rates post-pandemic, with the increases driven by the 65+ year age group alone (3.8% increase, 95% CI = 1.1–6.7, p = 0.027). Interestingly, in Malignant Neoplasm of the Bronchi, the opposite was seen, with the 65+ year group demonstrating a significant reduction in death rate post-pandemic (decrease of 4.1%, 95% CI -6.5 to −1.5, p = 0.017).

Both Dementia and Alzheimer's Disease and Ischaemic Heart Diseases mortality significantly increased post-pandemic in the 20–64 year age group, with no significant change in the 65+ age group (Table 1). All ITSA graphs are found in the *Appendix*.

## 4 Discussion

### 4.1 Key findings and context

In this study, publicly available data from the ONS was used to compare the trend in mortality rates of non-COVID conditions before and after the onset of the pandemic. Our research found that there were significant increases in the all-age mortality rates associated with: (1) Cirrhosis and Other Diseases of the Liver; (2) Symptoms Signs and Ill-defined Conditions; (3) Malignant Neoplasm of Breast. Interestingly we also found a significant decrease in the mortality rate associated with Chronic Lower Respiratory Disorders. In the conditions that did not show changes in all-age mortality rates, age-stratified analyses revealed differences in age-associated mortality.

Our research found there were more deaths during the studied pandemic period than were to be expected using pre-pandemic mortality trend estimates, which is consistent with previous studies. While research such as that by Vandoros et al. [6] reported increases in all-cause non-COVID mortality during the pandemic, our condition-specific mortality trends showed the pandemic to disproportionately affect specific conditions and age-groups.

Our results showed that ‘Cirrhosis and Other Diseases of the Liver’ (CDL) showed an increased all-age mortality rate during the pandemic; there are several potential explanations for this. Firstly, there are reports that COVID-19 can exacerbate liver cirrhosis progression and increase the mortality and morbidity of those with pre-existing cirrhosis [11]. Next, during the pandemic there was a reduction in organ donation and liver transplantation [12], and alcohol consumption also increased during the lockdowns [13], all of which could have exacerbated liver-related deaths. The average age of liver cirrhosis presentation is 51 ± 10 years [14], and our studies found the 20–64 age group suffered significant increases in mortality rate from these conditions whereas the 65+ age group did not. Perhaps resultingly this age group was more likely to be affected by the worsened conditions the pandemic reared for CDL.

Moreover, our study showed that ‘Symptoms Signs and Ill-defined Conditions’ had an increase in mortality trend from 2011 to 2020, which is consistent with previous reports of SSIDC-attributed deaths increasing [15]. Despite this trend there was a significant step jump from 2019 to 2020, suggesting the pandemic conditions had an additional impact on mortality. Spill-over deaths from COVID-19 may have contributed to this increase (e.g., COVID-related deaths that occurred more than 28 days after the positive test, which are not included in official figures), as could the incorrect reporting of patient's underlying cause of death which has been denoted previously [15]. The increased pressures that NHS staff were facing during the pandemic may have exacerbated this phenomenon. Furthermore, it has been widely reported that minority ethnic groups suffered greater severity from, and incidence of, COVID-19 [16]. Interestingly studies have shown that people from BAME communities are also more likely to be attributed SSIDC as an underlying cause of death than their white counterparts [17], which may have also propagated the 17% increase in mortality from 2019 to 2020.

While our study showed non-significant changes in all-age mortality associated with: (1) Cerebrovascular Disorders; (2) Ischaemic Heart Diseases; and (3) Dementia and Alzheimer's Disease, our age-stratified analyses of these conditions showed some striking similarities. All these conditions resulted in significant increases in their associated 20–64 age-group mortality rates and not their 65+ years mortality rates. The pandemic caused a multifaceted disruption to healthcare provision [18], and prioritising COVID-19 cases, especially in the elderly may have left non-COVID patients, and those younger, more vulnerable.

Regarding Cerebrovascular Disorders, studies have shown COVID-19 to be a risk factor for stroke. Cerebrovascular events are more common in those that have contracted COVID-19 [19], and COVID-19 positive patients suffer greater mortality from strokes [20]. Cerebrovascular disorders like stroke are generally viewed as diseases of the elderly; for that reason, it is theorised that younger patients are less likely to recognise stroke symptoms and are less likely to use emergency services for symptoms of stroke. Similarly, hospital care for strokes in younger patients is reportedly delayed due to the lack of recognition from healthcare teams [21]. Global reporting of a reduction in stroke admissions during the pandemic [22] may have worsened the impact of these factors in the 20–64 age-group and thus affected mortality.

Moreover, pre-existing cardiovascular disease has been associated with worse COVID-19 outcomes and increased risk of mortality, and reversely COVID-19 is known to induce myocardial injury [23]. There are reports that the COVID-19 lockdown increased cardiovascular risk factor development [24]. An increase in these risk factors coupled with a decrease in total admission and emergency department attendances after the lockdown commenced could be reasons for the increased mortality rate during the pandemic [25]. The issue of recognition could again be a potential reason as to why the 20–64 age-group were more significantly affected than the 65+ age-group.

With Dementia and Alzheimer's Disease, it has been widely reported that patients with dementia suffered greater severity of COVID-19 symptoms [26], but it has also been reported that COVID-19 infection can result in worsened dementia [27]. The isolating nature of the lockdown may have also caused further dementia deterioration [28]. Our research showed that the associated mortality rate had been increasing during the pre-pandemic years, with an overwhelming majority of Dementia and Alzheimer's Disease deaths coming from the 65+ age-group. Perhaps the added risk the COVID-19 pandemic caused was not substantial enough to confer additional mortality risk to those in this age group but was significant enough to impact mortality rates in the 20–64 age group.

The only group of conditions in our research that showed a significant decrease in all-age mortality rate during the pandemic were the Chronic Lower Respiratory Disorders. Previous research has shown that people with these disorders are at a greater risk of dying from COVID-19 [29], with conflicting reports as to how great the increased risk is [30]. The decreased mortality rate associated with these conditions during the pandemic could be due to these more vulnerable patients dying from COVID-19 instead of alternate lower respiratory conditions.

Finally, the neoplastic conditions we investigated were: (1) Malignant Neoplasm of Breast; (2) Malignant Neoplasm of Colon Sigmoid; and (3) Malignant Neoplasm of Trachea Bronchi. Discontinuation of breast and colon screening programmes during the pandemic produced fears that disruptions to diagnosis and early treatment would have repercussions for mortality rates downstream [31]. However, our research showed there to be a significant increase in the all-age mortality rate associated with Malignant Neoplasm of the Breast only in 2020 when compared to 2019. There was no significant change in all-age mortality rate associated with Malignant Neoplasm of Colon Sigmoid or Malignant Neoplasm of Trachea Bronchi.

Reports state that despite the disruptions caused by the pandemic and lockdowns, the number of patients receiving chemotherapy and surgery remained the same for colon cancer, even though outpatient volumes decreased [32]. The relatively undeterred treatment of these conditions could explain why the Malignant Neoplasm of Colon Sigmoid mortality rate showed no significant changes during the pandemic. Age-stratified analyses revealed however that although there was no significant change in the all-age mortality of Malignant Neoplasm of Trachea and Bronchi, there was a statistically significant decrease in mortality rate in the 65+ age-group. Lung cancer patients have a greater risk of mortality from COVID-19 [33], and perhaps the reduction is due to patients that would have otherwise died of lung cancer dying of COVID-19 instead.

### 4.2 Strengths and limitations

The study's strengths lie in utilising nationwide, routinely collected data from England and Wales to mitigate geographical selection bias. Interrupted time series analyses with a nine-year lead-in captures long-term trends, revealing the additional impact of COVID-19 and related measures. However, limitations include focusing on conditions with the highest death numbers from the ONS database, excluding many affecting the younger population. Stratifying data into broad age bands (20–64 and 65+) may overlook nuances, and more granular age-band analysis is recommended for future research.

Additionally, the study's design, comparing 2019 and 2020 mortality rates, lacks consideration for healthcare developments and other factors from 2011 to 2019. Unaccounted confounding factors, like deprivation, could influence pandemic death rates. We also defined 2020 as the pandemic period, despite the first case in England documented in late January 2020 and lockdown introduced in March 2020, thus the whole year of 2020 may not be fully considered as a full pandemic year, this may lead to an underestimate of the pandemic's true effects.

Moreover, an analysis of aggregated data is likely to have less power than a multilevel logistic regression analysis of the time series from individual sites. Since the data used here is national data, the confounding effect introduced by ‘place’ might have limited the power of the study. Lastly, while recognizing these strengths and limitations, we also acknowledge that further adjustment could have been made to account for the possibility of serial autocorrelation.

## 5 Conclusion

In this study, trends in non-COVID condition-specific mortality rates from 2011 to 2020 revealed that many non-COVID conditions were disproportionately affected during the pandemic period. Mortality rates of different age-groups within specific conditions were also differentially affected during the pandemic period. This may be due to the additional risk SARS-Cov-2 contraction had on these conditions or the effect the public health response had on condition-specific risk factor development and how healthcare was affected during the pandemic. Further work is required to understand the reasons behind these changes in mortality, and to develop interventions to address those drivers, so we are well-prepared to mitigate against excess deaths during this and future pandemics.

## Declaration of competing interest

There are no conflicts of interest to declare.

## Figures and Tables

**Figure:**
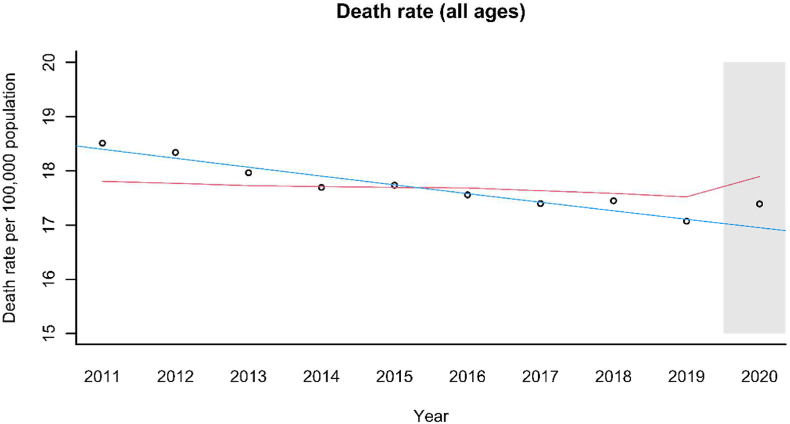


**Figure:**
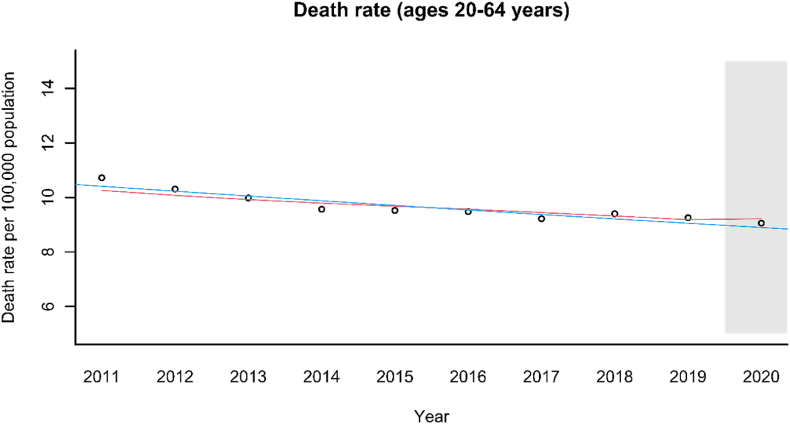


**Figure:**
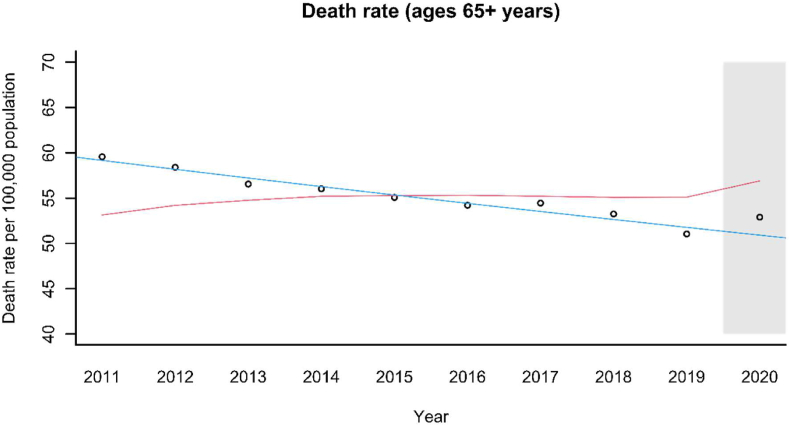


**Figure:**
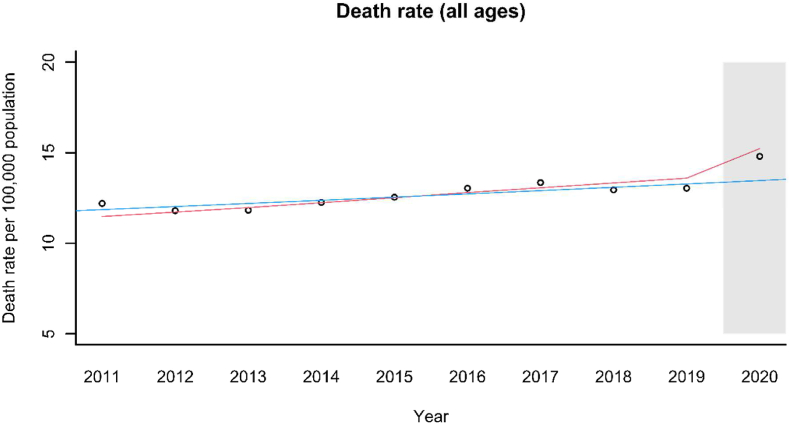


**Figure:**
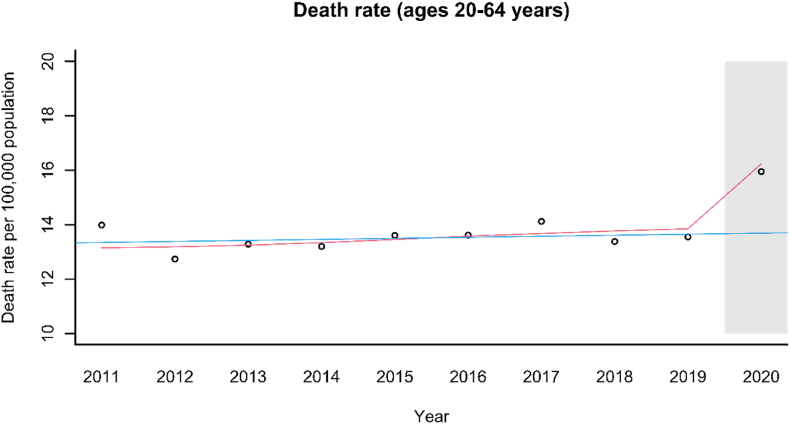


**Figure:**
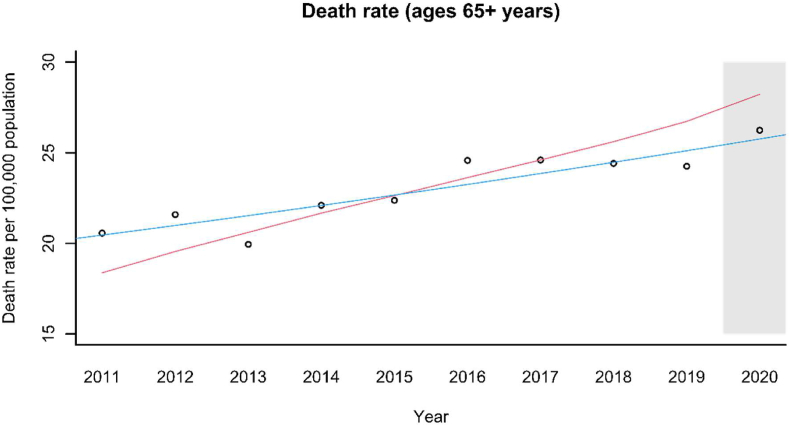


**Figure:**
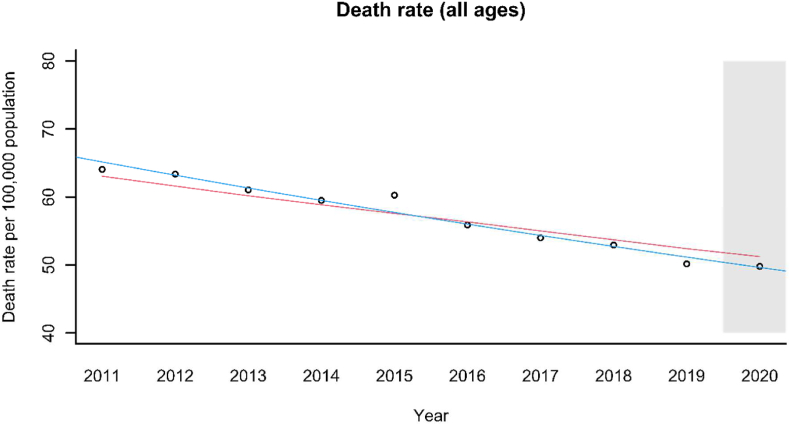


**Figure:**
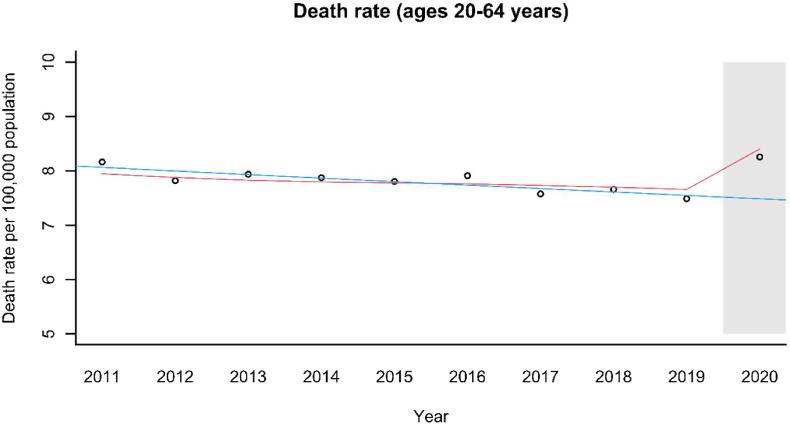


**Figure:**
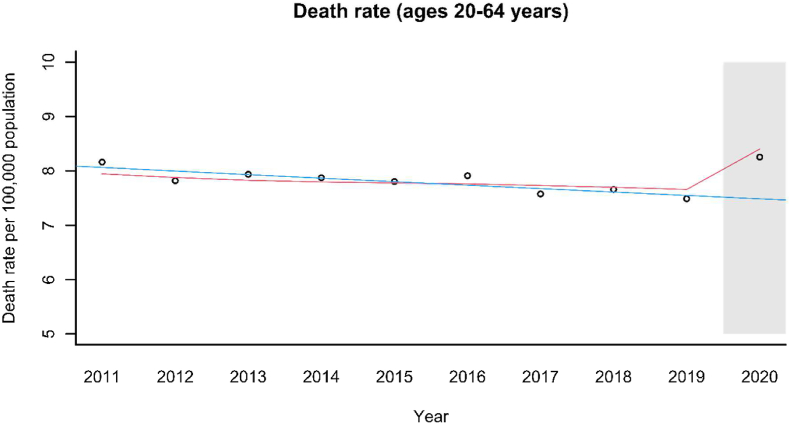


**Figure:**
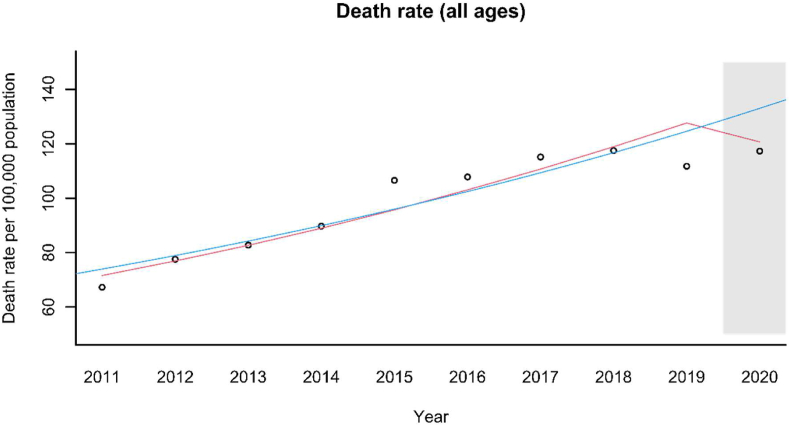


**Figure:**
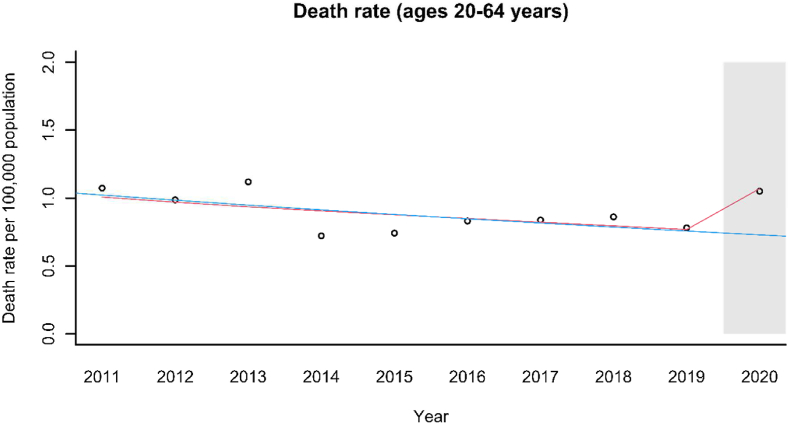


**Figure:**
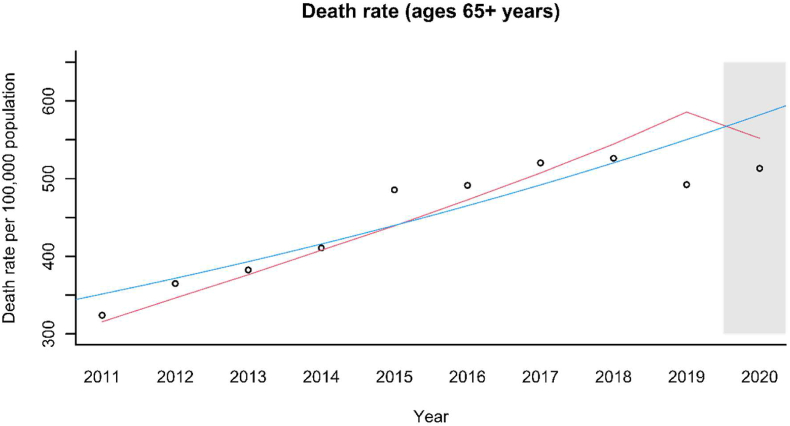


**Figure:**
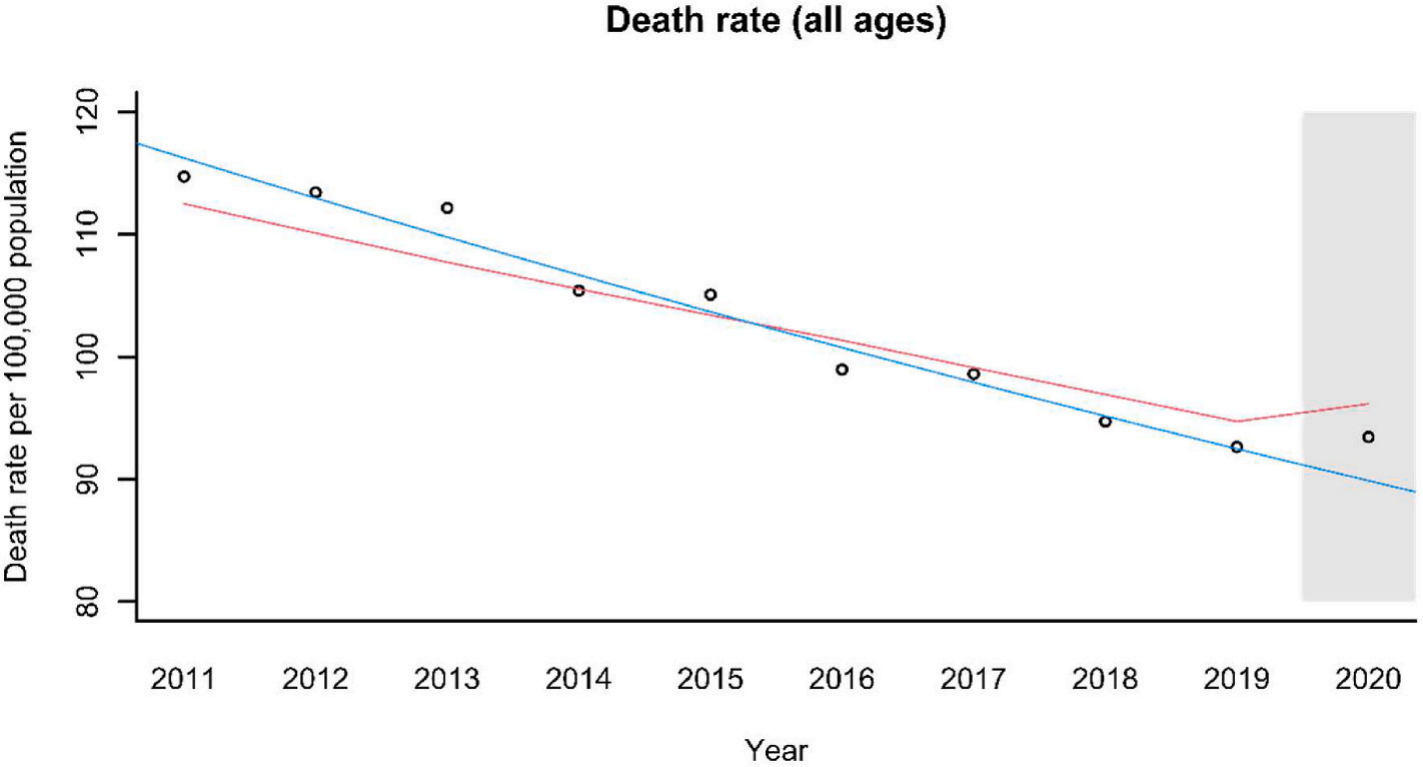


**Figure:**
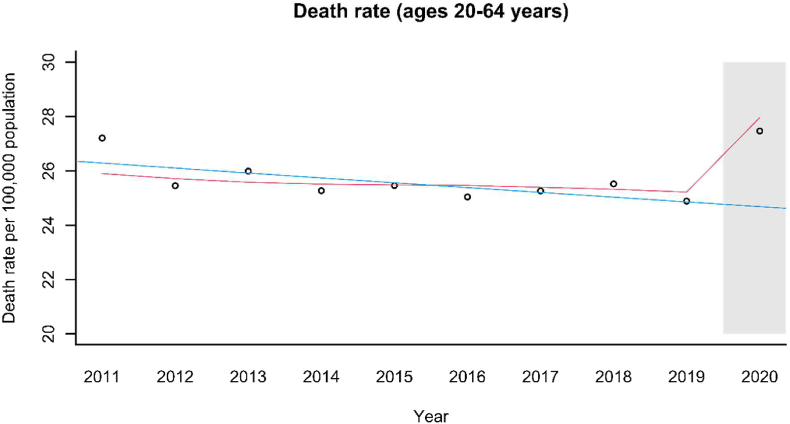


**Figure:**
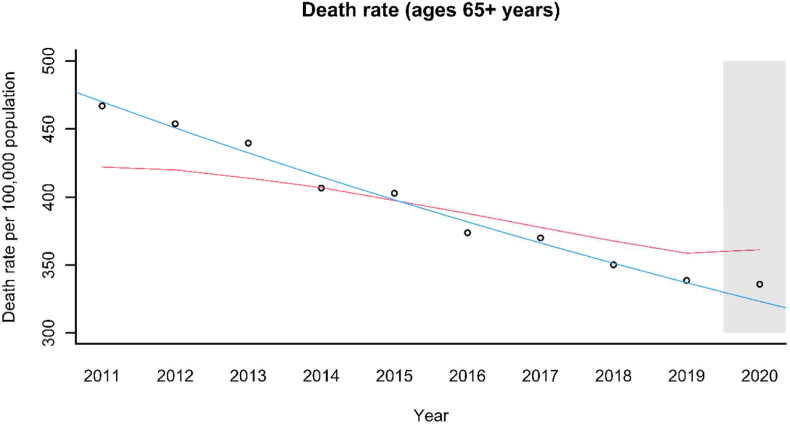


**Figure:**
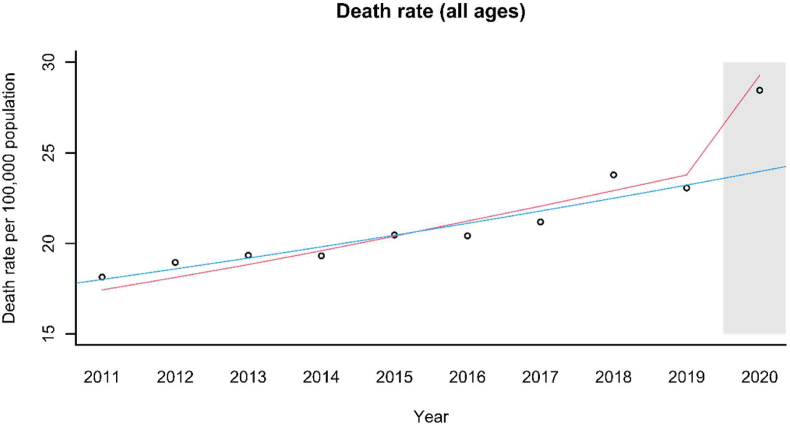


**Figure:**
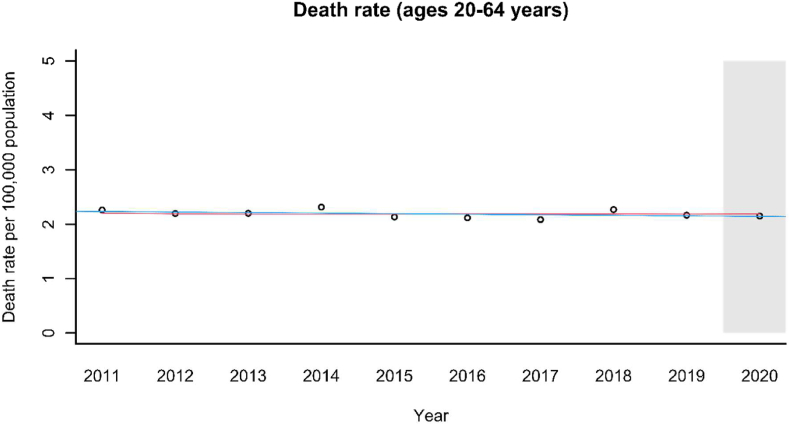


**Figure:**
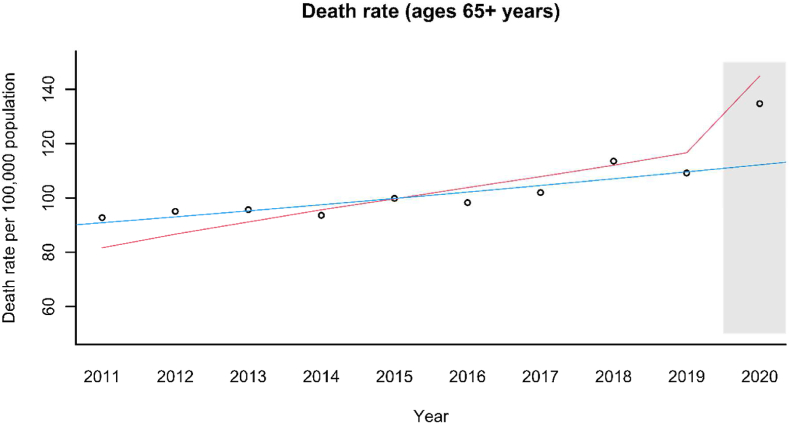


**Figure:**
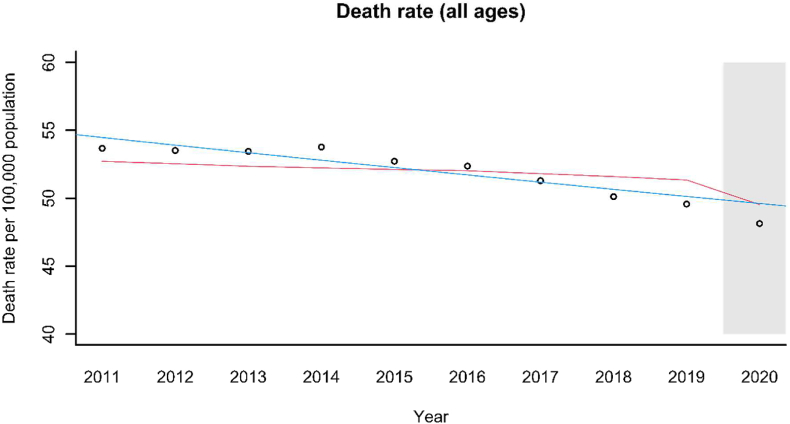


**Figure:**
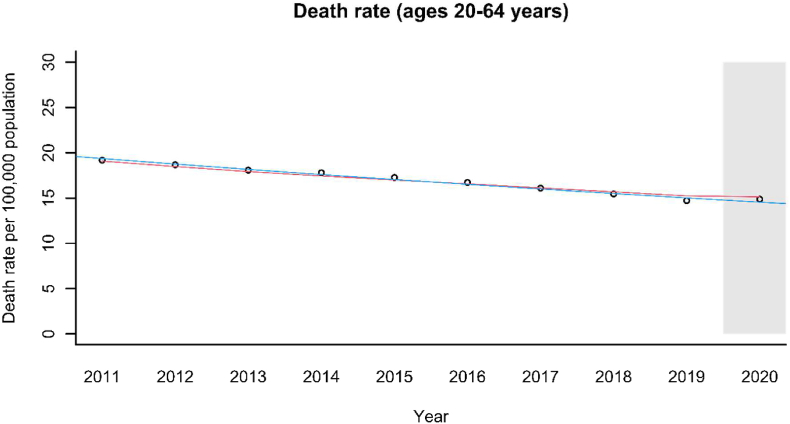


**Figure:**
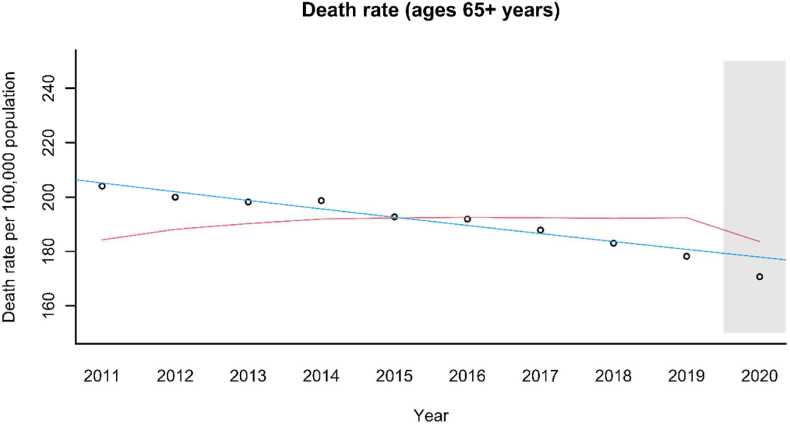


**Figure:**
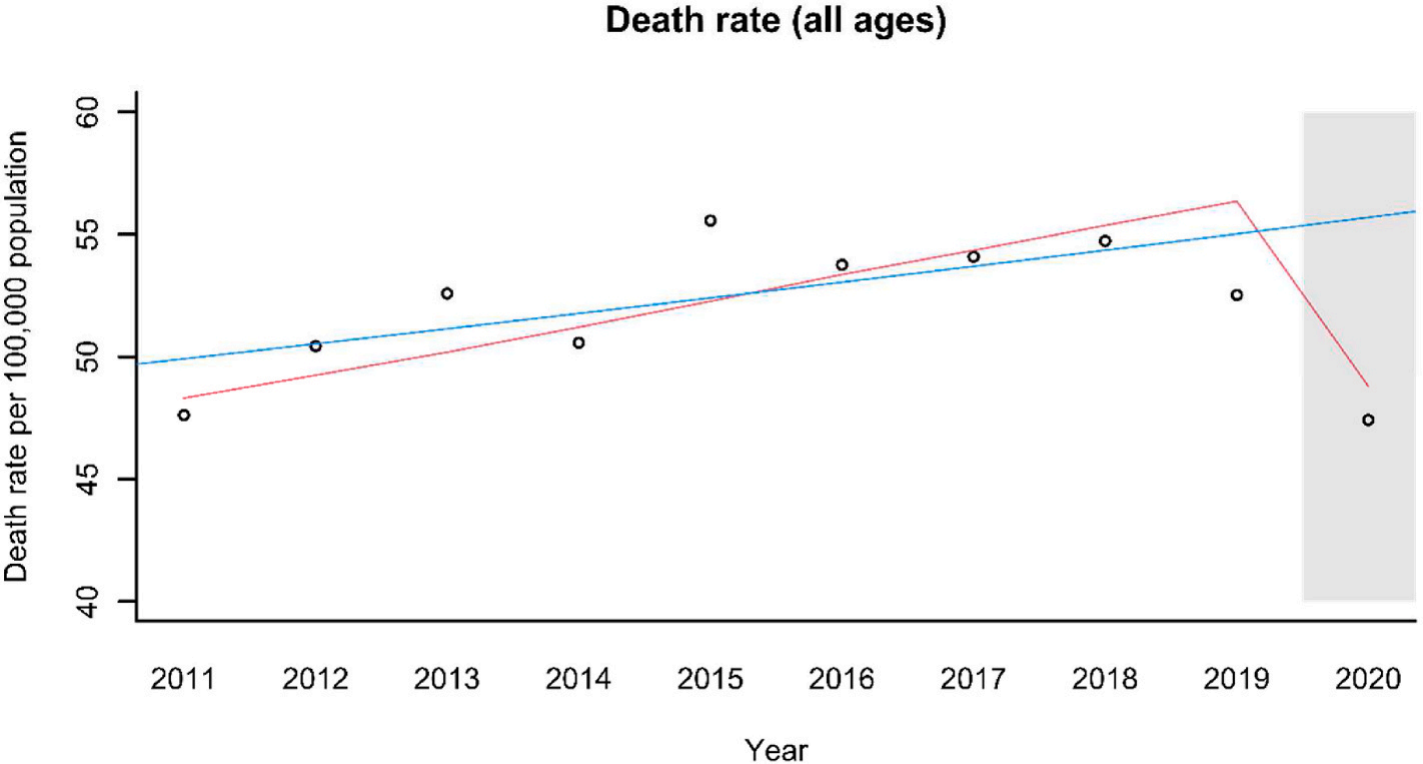


**Figure:**
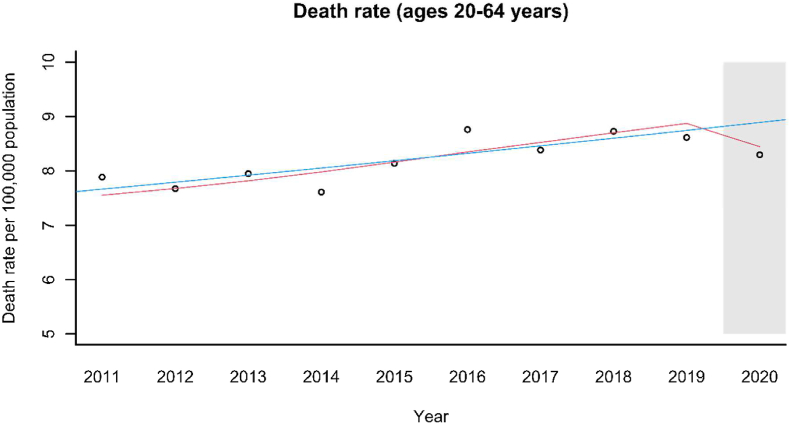


**Figure:**
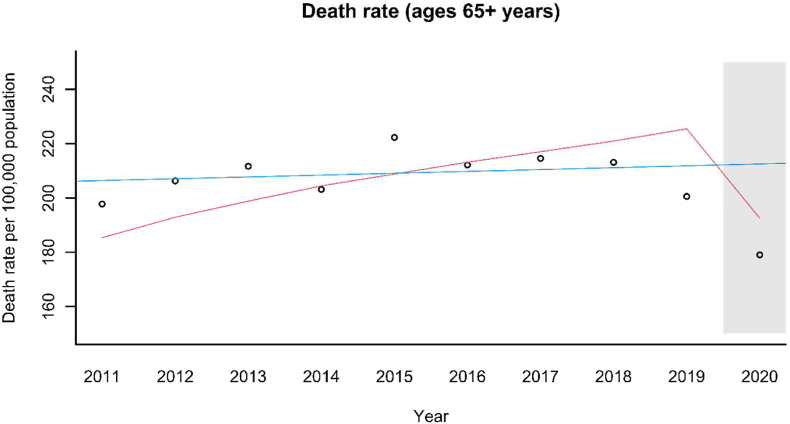


**Figure:**
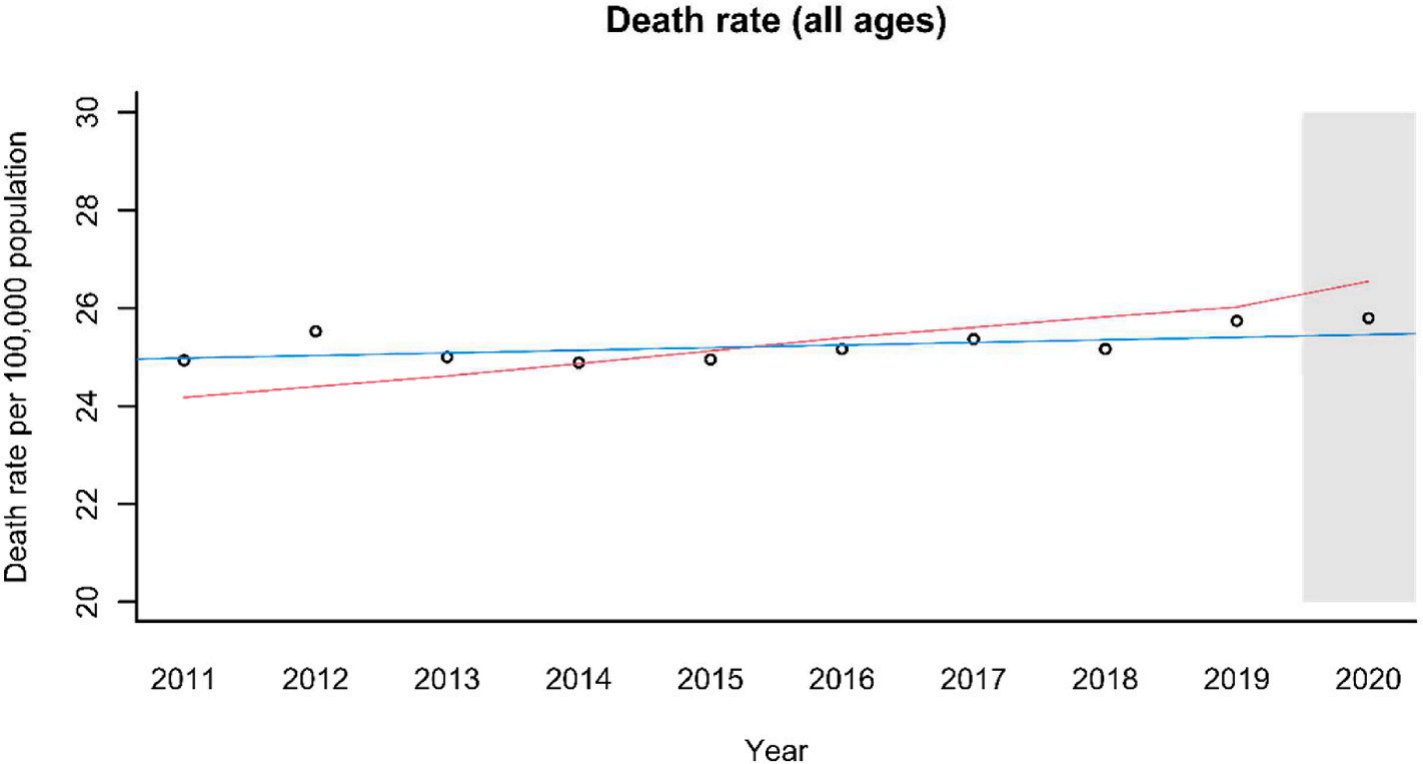


**Figure:**
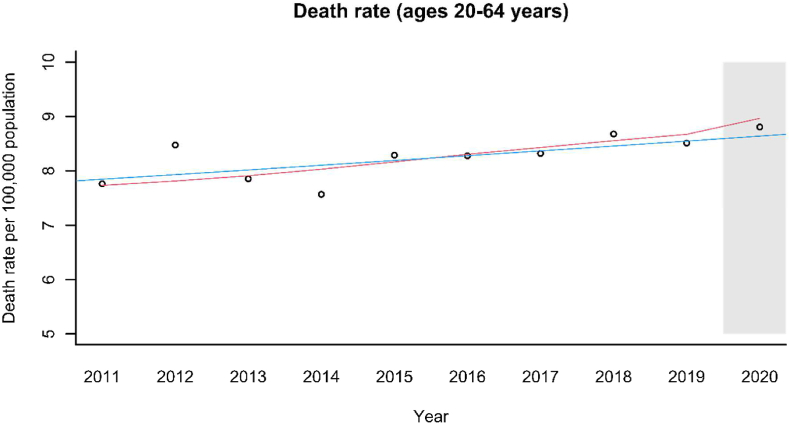


**Figure:**
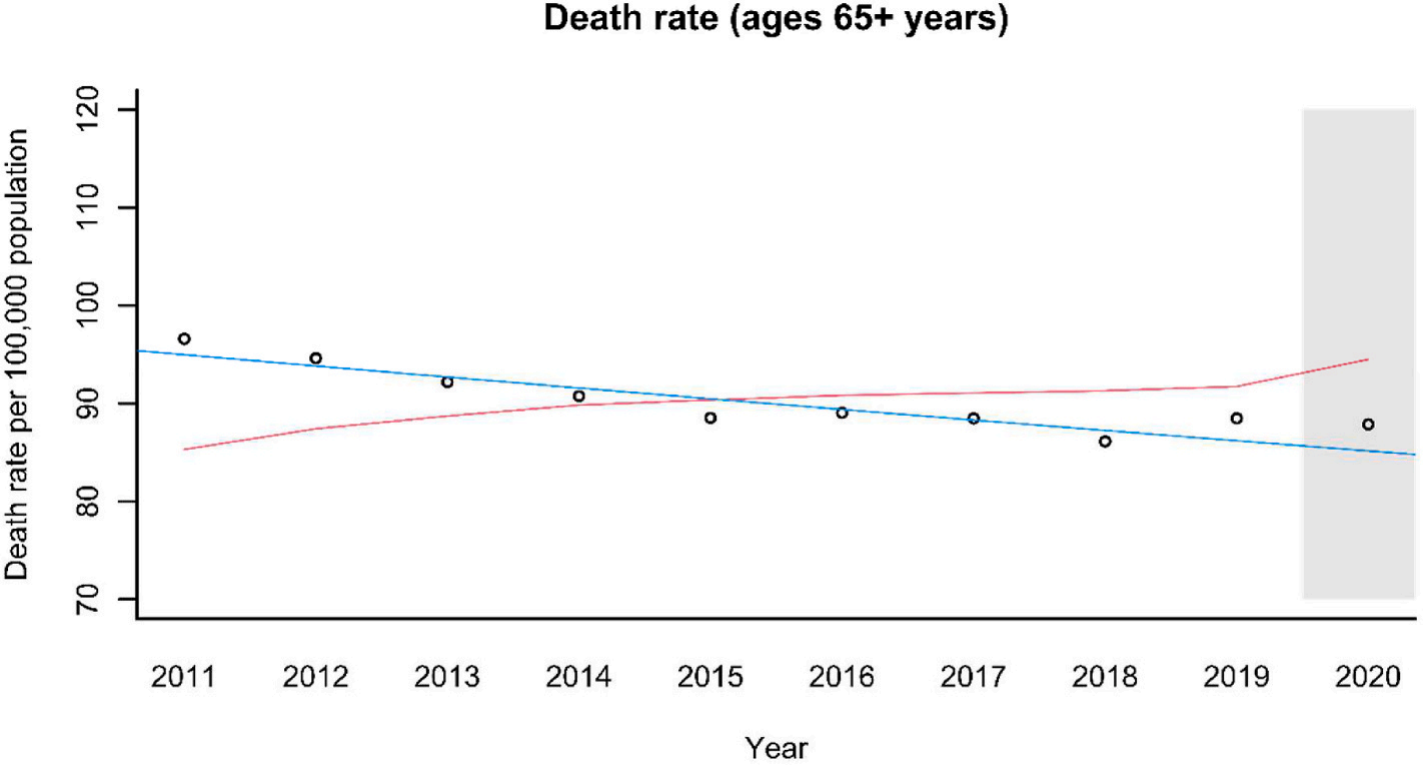


## Appendix All Interrupted Time Series Graphs

Grey box indicates the post-COVID period, red line is the true underlying trend, and blue line is the expected trend without COVID (the counterfactual).

### Malignant Neoplasm of Breast

All ages

Ages 20–64 years

Aged 65+ years

### Cirrhosis and Other Diseases of the Liver

All ages

Ages 20–64 years

Aged 65+ years

### Cerebrovascular Disorders

All ages

Ages 20–64 years

Aged 65+ years

### Dementia and Alzheimer's Disease

All ages

Ages 20–64 years

Aged 65+ years

### Ischaemic Heart Diseases

All ages

Ages 20–64 years

Aged 65+ years

### Symptoms Signs and Ill-defined Conditions

All ages

Ages 20–64 years

Aged 65+ years

### Malignant Neoplasm of Trachea Bronchi

All ages

Ages 20–64 years

Aged 65+ years

### Chronic Lower Respiratory Disorders

All ages

Ages 20–64 years

Aged 65+ years

### Malignant Neoplasm of Colon Sigmoid

All ages

Ages 20–64 years

Aged 65+ years

## References

[1] Rai P., Kumar B., Deekshit V., Karunasagar I., Karunasagar I. (2021). Detection technologies and recent developments in the diagnosis of COVID-19. Appl. Microbiol. Biotechnol..

[2] Cucinotta D., Vanelli M. (2020). WHO declared COVID-19 a pandemic. Acta Biol. Med. (Gdansk): Atenei Parmensis.

[3] WHO (2022). https://covid19.who.int.

[4] Islam N., Shkolnikov V., Acosta R., Klimkin I., Kawachi I., Irizarry R., Alicandro G., Khunti K., Yates T., Jdanov D., White M. (2021). Excess deaths associated with covid-19 pandemic in 2020: age and sex disaggregated time series analysis in 29 high income countries. BMJ.

[5] Vestergaard L., Nielsen J., Richter L., Schmid D., Bustos N., Braeye T., Dennisov G., Veideman T., Luomala O., Mottonen T., Fouillet A. (2020). Excess all-cause mortality during the COVID-19 pandemic in Europe-preliminary pooled estimates from the EuroMOMO network March to April 2020. Euro Surveill..

[6] Vandoros S. (2020). Excess mortality during the COVID-19 pandemic: early evidence from England and Wales. Soc. Sci. Med..

[7] Ioannidis J. (2020). Global perspective of COVID-19 epidemiology for a full-cycle pandemic. Eur. J. Clin. Invest..

[8] ONS (2022). https://www.ons.gov.uk/peoplepopulationandcommunity/birthsdeathsandmarriages/deaths/datalist?filter=datasets.

[9] Wolfe K., Sirota M., Clarke A. (2021). Age differences in COVID-19 risk-taking, and the relationship with risk attitude and numerical ability. R. Soc. Open Sci..

[10] O'Driscoll M., Ribeiro Dos Santos G., Wang L., Cummings D. (2021). Age-specific mortality and immunity patterns of SARS-CoV-2. Nature.

[11] Martinez M., Franco S. (2021). Impact of COVID-19 in liver disease progression. Hepatology Communications.

[12] Wang X., Lei J., Li Z., Yan L. (2021). Potential effects of coronaviruses on the liver: an update. Front. Med..

[13] Hepatology T. (2020). Drinking alone: COVID-19, lockdown and alcohol-related harm. The lancet. Gastroenterology and hepatology.

[14] Sajja K., Mohan D., Rockey D. (2014). Age and ethnicity in cirrhosis. J. Invest. Med..

[15] George J., Woodford H. (2008). Trends in hospital inpatient episodes for signs, symptoms and ill-defined condtions. Age Ageing.

[16] Hyder A., Hyder M., Nasir K., Ndebele P. (2021). Inequitable COVID-19 vaccine distribution and its effects. Bull. World Health Organ..

[17] Becker T., Wiggins C., Key C., Samei J. (1990). Symptoms, signs and ill-defined conditions: a leading cause of death among minorities. Am. J. Epidemiol..

[18] BMA (7 July 2023). https://www.bma.org.uk/advice-and-support/covid-19/what-the-bma-is-doing/covid-19-impact-of-the-pandemic-on-healthcare-delivery.

[19] Fraiman P., Junior C., Moro E., Cavallieri F., Zedde M. (2020). COVID-19 and cerebrovascular diseases: a systematic review and perspectives for stroke management. Front. Neurol..

[20] Tsivgoulis G., Palaiodimou L., Zand R., Lioutas V., Krogias C., Katsanos A., Shoamanesh A., Sharma V., Shahjouei S., Barachinni C., Vlachopoulos C. (2020). COVID-19 and cerebrovascular diseases: a comprehensive overview. Therapeutic Advances in Neurological Disorders.

[21] Singhal A., Biller J., Elkind M., Fullerton H., Jauch E., Kittner S., Levine D., Levine S. (2013). Recognition and management of stroke in young adults and adolescents. Neurology.

[22] Bass D., Meyer R., Barros G., Carroll K., Walker M., D'oria M., Levitt M. (2021). In Seminars in Vascular Surgery.

[23] Nishiga M., Wang D., Han Y., Lewis D., Wu J. (2020). COVID-19 and cardiovascular disease: from basic mechanisms to clinical perspectives. Nat. Rev. Cardiol..

[24] Muhammad D., Abubakar I. (2021). COVID-19 lockdown may increase cardiovascular disease risk factors. The Egyptian Heart Journal.

[25] Ball S., Banerjee A., Berry C., Boyle J., Bray B., Bradlow W., Chaudhry A., Crawley R., Danesh J., Denniston A., Falter F. (2020). Monitoring indirect impact of COVID-19 pandemic on services for cardiovascular diseases in the UK. Heart.

[26] Wang Q., Davis P., Gurney M., Xu R. (2021). COVID-19 and dementia: analyses of risk, disparity, and outcomes from electronic health records in the US. Alzheimer’s Dementia.

[27] Alzheimer's Society (2021). https://www.alzheimers.org.uk/get-support/coronavirus/dementia-risk.

[28] Tuijt R., Frost R., Wilcock J., Robinson L., Manthorpe J., Rait G., Walters K. (2021). Life under lockdown and social restrictions - the experiences of people living with dementia and their carers during the COVID-19 pandemic in England. BMC Geriatr..

[29] Beltramo G., Cottenet J., Mariet A., Georges M., Piroth L., Tuber-Bitter P., Bonniaud P., Quantin C. (2021). Chronic respiratory diseases are predictors of severe outcome in COVID-19 hospitalised patients: a nationwide study. Eur. Respir. J..

[30] Aveyard P., Gao M., Lindson N., Hartmann-Boyce J., Watkinson P., Young D., Coupland C., San Tan P., Clift A., Harrison D., Gould D. (2021). Association between pre-existing respiratory disease and its treatment, and severe COVID-19: a population cohort study. Lancet Respir. Med..

[31] Turnbull C. (2021). Effect of COVID-19 on colorectal cancer care in England. The Lancet Gastroenterology and Hepatology.

[32] Xu Y., Huang Z., Zheng C., Li C., Zhang Y., Guo T., Liu F., Xu Y. (2021). The impact of COVID-19 pandemic on colorectal cancer patients: a single-centre retrospective study. BMC Gastroenterol..

[33] Passaro A., Bestvina C., Velez M., Garassino M., Garon E., Peters S. (2021). Severity of COVID-19 in patients with lung cancer: evidence and challenges. Journal for Immunotherapy of Cancer.

